# Identification of SNPs and Candidate Genes Associated With Salt Tolerance at the Seedling Stage in Cotton (*Gossypium hirsutum* L.)

**DOI:** 10.3389/fpls.2018.01011

**Published:** 2018-07-11

**Authors:** Zhengwen Sun, Hanli Li, Yan Zhang, Zhikun Li, Huifeng Ke, Liqiang Wu, Guiyin Zhang, Xingfen Wang, Zhiying Ma

**Affiliations:** North China Key Laboratory for Crop Germplasm Resources of Education Ministry, Key Laboratory for Crop Germplasm Resources of Hebei Province, Hebei Agricultural University, Baoding, China

**Keywords:** cotton, salt tolerance, seedling, genome-wide association study, SNP, candidate genes

## Abstract

Salt tolerance in cotton is highly imperative for improvement in the response to decreasing farmland and soil salinization. However, little is known about the genetic basis underlying salt tolerance in cotton, especially the seedling stage. In this study, we evaluated two salt-tolerance-related traits of a natural population comprising 713 upland cotton (*Gossypium hirsutum* L.) accessions worldwide at the seedling stage and performed a genome-wide association study (GWAS) to identify marker-trait associations under salt stress using the Illumina Infinium CottonSNP63K array. A total of 23 single nucleotide polymorphisms (SNPs) that represented seven genomic regions on chromosomes A01, A10, D02, D08, D09, D10, and D11 were significantly associated with the two salt-tolerance-related traits, relative survival rate (RSR) and salt tolerance level (STL). Of these, the two SNPs i46598Gh and i47388Gh on D09 were simultaneously associated with the two traits. Based on all loci, we screened 280 possible candidate genes showing different expression levels under salt stress. Most of these genes were involved in transcription factors, transporters and enzymes and were previously reported as being involved in plant salt tolerance, such as *NAC, MYB, NXH, WD40, CDPK, LEA*, and *CIPK*. We further validated six putative candidate genes by qRT-PCR and found a differential expression level between salt-tolerant and salt-sensitive varieties. Our findings provide valuable information for enhancing the understanding of complicated mechanisms of salt tolerance in *G. hirsutum* seedlings and cotton salt tolerance breeding by molecular marker-assisted selection.

## Introduction

Soil salinity is a major abiotic stress that threatens crop yield, the ecological environment and agricultural sustainability (Hamwieh et al., 2011; Farooq et al., 2017). Indeed, salinity almost affected one billion hectares of arid and semi-arid areas globally (Dagar and Minhas, 2016), and soil salinity will become progressively more severe over time due to climatic changes, unscientific irrigation and excessive fertilization (Li et al., 2008; Han J. et al., 2015). Salt salinity is predicted to affect more than 50% of all arable land by 2050 (Ashraf and Wu, 1994; Demiral and Türkan, 2006).

Cotton (*Gossypium hirsutum* L., AADD, 2*n* = 52) is an economically important crop worldwide and can be used for soil reclamation as a pioneer crop of saline-alkali land, which provides improved farmland for grain crops production (Huang et al., 2013). Meanwhile, the demand for cotton fiber production will increase as the human population grows. To meet this challenge, breeders are working on developing new varieties using conventional and modern breeding methods to enhance cotton yield and tolerance under salt conditions. First, it is important to dissect the complex and dynamic mechanisms of plants under saline stress. Salinity affects plants in osmotic and ionic ways. The osmotic component of salinity leads to the reduction of water transport and partial stomatal closure. Interestingly, plants temporarily adapt to saline conditions using K^+^, Na^+^ and osmoprotectants for osmoregulation (Le et al., 1984; Munns and Tester, 2008). However, under long-term salt exposure, plants are affected by the continuing osmotic damage plus the excessive accumulation of Na^+^ in plant cells. High concentrations of Na^+^ inhibit enzyme activities and compete against K^+^ which is particularly important for enzyme functions (Bertorello et al., 1991; Tester and Davenport, 2003). To counter ionic toxicity, plants have mechanisms to compartmentalize Na^+^ in vacuoles and to remove the excess of Na^+^ from the cytosol to the apoplast (Maathuis and Amtmann, 1999; Munns et al., 2012).

Conventionally, Quantitative trait locus (QTL) mapping of salt-related traits has become an effective approach, which measures associations between genetic markers and traits using bi-parental populations. In the past few years, QTL mapping has been used for many plants using different statistical models, genetic markers and crossed populations (Mano and Takeda, 1997; Wang et al., 2012; Xu et al., 2012; Oyiga et al., 2017). In cotton, there have been only a few studies addressing salt tolerance traits (Tiwari et al., 2013; Oluoch et al., 2016). However, the constraint to QTL mapping is that QTLs are located in big genomic regions and contain too many genes, and genetic diversity between the parents and recombination progeny is limited. Moreover, backcrossing may take months or even years (Weigel, 2012).

Genome-wide association studies based on linkage disequilibrium (LD) are an alternative tool for studying the associations between phenotype and genotype. Compared to traditional QTL mapping, GWAS could use SNPs that are positioned across the genome as molecular markers for dissecting complex traits (Si et al., 2016). Moreover, GWAS can handle up to several million SNP markers and thousands of natural accessions as a mapping population (Huang et al., 2010; Chen et al., 2014). GWAS has been successfully applied in rice, Arabidopsis, maize, wheat, barley and other crops and contributed to identifying SNP markers that are associated with a trait of interest across diverse natural accessions (Atwell et al., 2010; Xue et al., 2013; Chen et al., 2016; McCouch et al., 2016). In cotton, association analysis had been adopted for important target traits, but all based on SSR markers (Abdurakhmonov et al., 2008; Mei et al., 2013; Cai et al., 2014; Jia et al., 2014b; Qin et al., 2015; Nie et al., 2016). Recently, with the development of SNP arrays, genotyping and sequencing technologies, some GWAS studies have been successfully applied for detecting genetic variations underlying diverse complex traits such as flowering time, yield, fiber quality and resistance to Verticillium wilt of cotton (Su et al., 2016c; Huang et al., 2017; Li et al., 2017; Sun et al., 2017). However, only few studies have applied a GWAS strategy of large-scale cotton accessions and SNP markers to unravel the molecular mechanism for salt tolerance (Diouf et al., 2017).

In the current study, we performed GWAS for the relative survival rate (RSR) and salt tolerance level (STL) with 10,511 SNPs at seedling stage under salt stress in 713 *G. hirsutum* accessions. The objectives of this study were to identify the associated SNPs and candidate genes, to contribute to understanding the mechanisms for salt tolerance and to develop molecular markers for accelerating breeding to enhance salt tolerance in cotton.

## Materials and Methods

### Cotton Germplasm

The association panel of 713 upland cottons accessions was used in this study derived from the recently published study (Sun et al., 2017). These germplasms were collected from different regions of China (585) and other countries (128) including the United States of America and Australia (Supplementary Table S1).

### Evaluation of Salt Tolerance and Phenotypic Data Analysis

After the cotton seeds were delinted by sulfuric acid, 400 healthy and full seeds were selected from each germplasm accession. Each accession was set to three replicates and one water control treatment, and each replicate and water treatment contained 100 seeds. The seeds were placed evenly in a germination box containing 800 g dry quartz sand and covered evenly with 250 g dry quartz sand above the seeds, eventually adding 250 mL of 0.3% saline solution. The germination boxes were placed in a constant-temperature chamber at 28°C for 7 days with a 12/12-h light/dark regimen during the period without any treatment. We counted the number of surviving seedlings after sowing for 7 days and calculated the survival rate. Because the vitality of the seeds themselves will bring the error, it is necessary to calculate the RSR. Using a two-factor test design for optimal NaCl concentration, the A factor was the salt content (refers to the weight percentage of NaCl on dry quartz sand) with the above five different concentrations (0.1, 0.2, 0.3, 0.4, and 0.5%); the B factor was the control varieties containing the salt-tolerant cultivar Yu2067 and salt-sensitive cultivar Lumianyan21. The two cultivars were treated with five different NaCl concentrations. The RSR of the two cultivars had no difference when the NaCl concentration was 0.1 and 0.2% and the salt-sensitive cultivar Lumianyan21 had almost no survival when the NaCl concentrations were 0.4 and 0.5%. The results showed that the 0.3% NaCl content was suitable to evaluate the salt tolerance of the cotton seedlings.

The evaluated salt-tolerant traits included the RSR [RSR % = SR/control SR × 100, where SR % = the number of survival seedlings/total seed number used in the test × 100], and STL [STL was divided into four grades (from 1 to 4) according to the RSR; 4 represents salt sensitivity (0–49.9%), 3 represents salt tolerance (50.0–74.9%), 2 represents salt resistance (75.0–89.9%), and 1 represents high salt resistance (>90%)]. Statistical analysis of the RSR and STL was performed in SPSS 22.0. The frequency distribution of each trait and descriptive statistics were performed using all the phenotypic data from 713 cotton accessions.

### Genome-Wide Association Analysis

Population structure, relative kinship and LD analysis had already been analyzed in a previous study (Sun et al., 2017). Here, association analysis was conducted using the Q + K model with a total of 10,511 SNPs [call rate > 85% and minor allele frequency (MAF) > 0.05] from the Illumina CottonSNP63K array (Hulse-Kemp et al., 2015) and the Q + K model, which was implemented via a mixed linear model (MLM) in TASSEL 3.0 (Bradbury et al., 2007). According to the Bonferroni correction principle, -log_10_ (*P*) > 3.97 (*P* = 1/*n, n* is the SNP numbers in this study) should be as threshold, but the Bonferroni correction is too stringent, we can’t almost identify the significant SNPs for two traits with this threshold. To obtain more associated SNPs, the significantly associated SNP markers with salt-tolerant-related traits were identified according to -log_10_ *P* > 3.0 (Hatzig et al., 2015; Zhang et al., 2015a).

### Identification of Candidate Genes

The genes within 200 kb near significant SNP loci were assigned as putative candidates based on the gene annotation in the *G. hirsutum* TM-1 genome (Zhang et al., 2015b). The interval of 200 kb was considering the LD decay distances and in comparison with other crops (Sun et al., 2017). GO enrichment and KEGG pathway analysis were carried out for all candidate genes (Xie et al., 2011). To further screen the possible candidate genes involved in the salt response, these genes were analyzed using the expression level of the seedlings at 1, 3, 6, and 12 h under 400 mM salt concentration reported by Zhang et al. (2015b).

### Expression Profile of Putative Candidate Genes

To validate the results of the GWAS, five salt-tolerant and five salt-sensitive varieties were selected for gene expression analysis of the putative candidates by qRT-PCR (Supplementary Table S6). Plants were grown in germination boxes containing quartz sand for 7 days with 0.3% NaCl content. Roots were sampled and immediately frozen in liquid nitrogen and stored at -80°C for RNA extraction. Total cDNA was synthesized with PrimeScript^TM^ RT reagent Kit (Perfect Real Time) (TaKaRa). The qRT-PCR reactions contained 10 μL of SYBR Premix DimerEraser (TaKaRa), 2.0 μL of cDNA, 0.6 μL of primer, and ddH_2_O to a final volume of 20 μL. The reactions were amplified for 30 s at 95°C, followed by 40 cycles of 95°C for 5 s, 55°C for 30 s and 72°C for 30 s. All reactions were performed in three independent biological replicates, each with three technical replicates, using the Roche LightCycler96 Real-Time PCR System. The primers are listed in Supplementary Table S7. *GhUBQ*14 expression was used as the internal control for qRT-PCR. Relative gene expression values were calculated using the 2^-ΔCT^ method (Schmittgen and Livak, 2008).

### Determination of Physiological and Biochemical Indexes

A salt-tolerant cultivar (Suwu77-702) and a salt-sensitive cultivar (Shann424) were selected to grow for 7 days in a germination box, and then moved into 1/2 Hogland nutrient solution until the three-true-leaf period (about another 14 days). Under 200 mmol/L NaCl stress, leaves were sampled at 0, 3, 6, 8, 12, 24, and 48 h for measuring the content of the protein, POD, MDA, and H_2_O_2_ (Liu and Li, 2007; Cai et al., 2017). Three biological replicates were performed.

## Results

### Phenotypic Variation for Salt-Tolerant Traits Among Accessions

**FIGURE 1:**
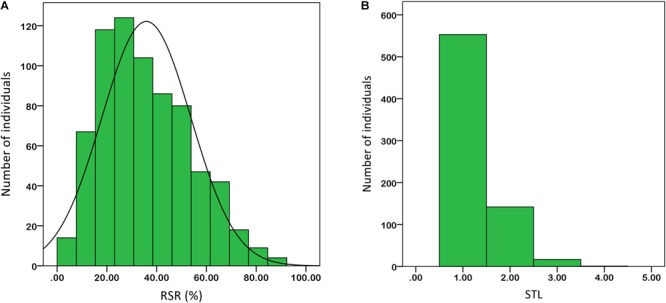
Phenotypic diversity of the two salt-tolerance-related traits in 713 cotton accessions based on 0.3% salt treatment. **(A)** RSR. **(B)** STL. Data were averaged over three replicates.

Under salt stress of 0.3% NaCl content, extensive phenotypic variations were observed for RSR and STL in 713 upland cotton accessions. The RSR ranged from 3.38 to 92.06% with an average of 35.85%, and the STL ranged from 1.00 to 4.00 with an average of 1.25. The coefficient of variation (CV) was 49.96 and 39.52% (**Table 1**). Of the 713 accessions, 160 were identified as salt-tolerant, and 18 were salt-resistant in which Litai8 had 92.06% for RSR and belonged to a high salt-resistant level according to the STL standard. The phenotypic distribution of RSR and STL showed continuous variation (**Figure 1**), while RSR was approximately a normal distribution, indicating that these two traits were quantitative traits controlled by multiple genes.

**Table 1:** Phenotypic variation for the two salt-tolerance traits under salt treatment in the panel of cotton accessions.

Trait	Max	Min	Mean	*SD*	Kurtosis	Skewness	CV (%)
RSR	92.06	3.38	35.85	17.91	-0.35	0.56	49.96
STL	4.00	1.00	1.25	0.49	3.22	1.89	39.52

*RSR, relative survival rate; STL, salt tolerance level; Max, maximum; Min, minimum; SD, standard deviation; CV (%), coefficient of variation.*

### Association Mapping Between SNPs and Two Salt-Tolerant Traits

**FIGURE 2:**
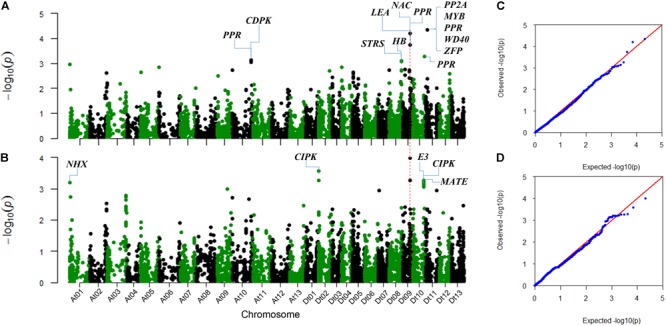
Manhattan and QQ plots for the two salt-tolerance-related traits. **(A)** Manhattan plot for RSR. **(B)** Manhattan plot for STL. **(C)** QQ plot for RSR. **(D)** QQ plot for STL. The corresponding *Arabidopsis thaliana* homologous genes linked to the significant SNPs are shown.

The SNP markers associated with the two salt-tolerant related traits, RSR and STL, were identified using the MLM model considering population structure (Q) and kinship (K) in TASSEL 3.0 software (**Figure 2**). We identified a total of 23 significantly associated SNPs for the two traits. These SNPs were distributed on chromosomes A01, A10, D02, D08, D09, D10, and D11 (**Table 2**).

**Table 2:** List of SNP markers significantly associated with RSR and STL in the upland cotton association panel.

Traits	SNP	Chr.	Position	Alleles	MAF	-log_10_ *P*	*R*^2^ (%)
RSR	i28278Gh	A10	92028950	A/G	0.09	3.03	1.93
	i31650Gh	A10	92029662	A/G	0.09	3.03	1.93
	i28055Gh	A10	92136937	A/G	0.07	3.13	1.99
	i31466Gh	D08	58340490	G/T	0.18	3.09	1.96
	i04513Gh	D08	58342391	A/G	0.18	3.09	1.96
	i38423Gh	D08	58344064	G/A	0.18	3.09	1.97
	i46598Gh	D09	35026265	G/A	0.32	4.20	2.70
	i47388Gh	D09	35027832	T/C	0.31	3.73	2.38
	i12146Gh	D10	56236487	G/A	0.08	3.26	2.08
	i06916Gh	D11	5993674	G/A	0.22	4.35	2.78
STL	i46938Gh	A01	2377299	C/T	0.28	3.19	2.06
	i04851Gh	D02	548563	C/T	0.13	3.27	2.11
	i47348Gh	D02	578006	T/C	0.07	3.57	2.31
	i46598Gh	D09	35026265	G/A	0.32	3.99	2.60
	i47388Gh	D09	35027832	T/C	0.31	3.27	2.11
	i12076Gh	D10	51426932	C/T	0.31	3.22	2.08
	i60613Gb	D10	51430332	G/A	0.31	3.26	2.10
	i42017Gh	D10	52624958	G/A	0.47	3.07	1.99
	i24986Gh	D10	52684268	C/T	0.46	3.20	2.06
	i33471Gh	D10	52710803	A/G	0.46	3.13	2.02
	i20955Gh	D10	52740721	A/G	0.46	3.13	2.03
	i40669Gh	D10	52741391	T/C	0.46	3.13	2.02
	i43909Gh	D10	52797907	A/C	0.46	3.13	2.02
	i22025Gh	D10	52803468	G/A	0.46	3.20	2.06
	i29606Gh	D10	52803527	A/G	0.46	3.19	2.06

For RSR, 10 associated SNPs were identified on chromosomes A10, D08, D09, D10, and D11 (**Table 2**). The phenotypic variation explained by a single SNP ranged from 1.93 to 2.78%, and the strongest association locus (i06916Gh) was found at 5.99 Mb on D11 (**Figure 2A**).

For STL, a total of 15 association signals were detected and distributed on chromosomes A01, D02, D09, and D10, explaining 1.99–2.60% of the phenotypic variation (**Table 2**). Among them, the strongest association signal (i46598Gh) was found at 35.02 Mb on D09. Notably, the SNP i46598Gh and i47388Gh on D09 were significantly associated with both STL and RSR. In addition, a GWAS peak containing 10 SNPs was detected on chromosome D10 (**Figure 2B**), of which eight SNPs were closed in the chromosome (**Table 2**).

### Prediction of Salt-Tolerant Candidate Genes

To predict the potential candidate genes related to RSR and STL, we identified genes located within 200 kb in the genome according to the physical position of each significant locus and the functional annotation of the homologous genes in Arabidopsis. Based on the *G. hirsutum* TM-1 reference genome, we obtained 280 candidate genes associated with the two traits (Supplementary Table S2). GO enrichment and KEGG pathway analysis were conducted for all candidate genes. Tetrahydrofolate interconversion (GO: 0035999) with four genes and the regulation of the phenylpropanoid metabolic process (GO: 2000762) with four genes were the categories most significantly enriched (Supplementary Table S3). These genes were in the amino acid dehydrogenase family protein and peroxidase superfamily protein. Meanwhile, there were three genes enriched significantly in oxidoreductase activity (GO: 0016646). For the KEGG pathway, the top significantly enriched pathway was monoterpenoid biosynthesis with six genes, which was part of the NAD (P)-binding Rossmann-fold superfamily of proteins (Supplementary Table S3).

**FIGURE 3:**
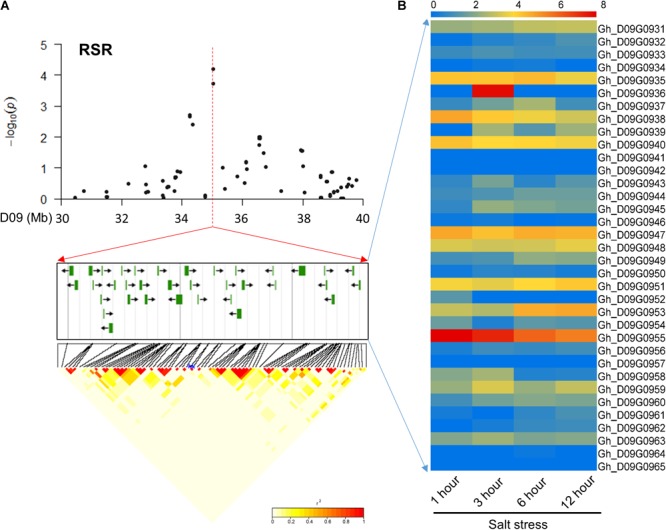
Identification of the candidate genes for RSR and STL on the peak of chromosome D09. **(A)** Local Manhattan plot (top) and LD heatmap (bottom) surrounding the peak. The 35 candidate genes were presented in the middle. **(B)** Heat map of the expression of the 35 candidate genes at four time points. The samples were collected at 1, 3, 6, and 12 h under salt stress.

In chromosome D09, 35 common candidate genes were identified for the RSR and STL surrounding peak SNPs (i46598Gh and i47388Gh) with high LD (**Figure 3A**). These candidate genes were involved in different transcription factors, binding proteins, membrane transport proteins and other proteins of unknown function. Further, these candidate genes were screened using transcriptome sequencing data^1^. The results showed that the most of the genes had varying degrees of expression at four time points (1, 3, 6, and 12 h) under salt stress (**Figure 3B**). Among them, *Gh_D09G0943* and *Gh_D09G0950* are orthologous to the genes *NAC061* and *NAC089*, respectively, and *Gh_D09G0958* and *Gh_D09G0959* are *LEA* genes (Supplementary Table S4). These genes could be promising candidates according to previous reports of salt-related genes in other plants (Puranik et al., 2012; Hu et al., 2016).

**FIGURE 4:**
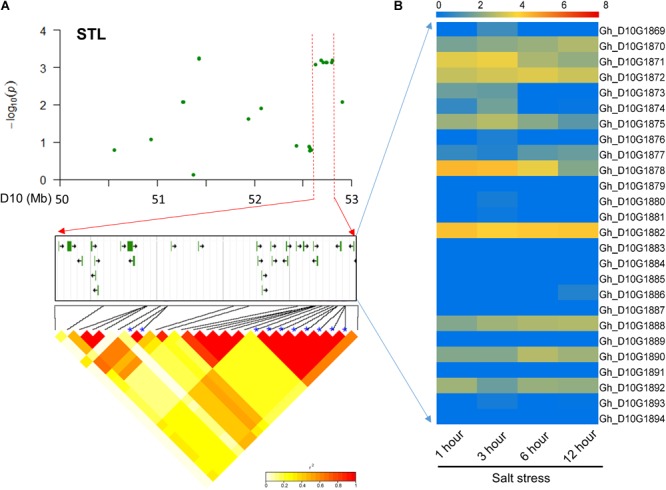
Identification of the candidate genes for STL on the peak of chromosome D10. **(A)** Local Manhattan plot (top) and LD heatmap (bottom) surrounding the peak. The 26 candidate genes were presented in the middle. **(B)** Heat map of the expression of the 26 candidate genes at four time points. The samples were collected at 1, 3, 6, and 12 h under salt stress.

For STL, 26 candidate genes surrounding the eight significant SNP loci with high LD were found on chromosome D10 (**Figure 4A**). Most of these genes had preferential expression at different time points with salt treatment (Supplementary Table S5). Among these genes, *Gh_D10G1882* and *Gh_D10G1888* were preferentially expressed and *Gh_D10G1874* had higher expression at 3 h (**Figure 4B**). The above three genes were annotated as peroxidase, E3 ubiquitin-protein ligase gene and CBL-interacting protein kinase, respectively, which were involved and functioned in plant responses to salt stress (Yee and Goring, 2009; Jin et al., 2016).

### Gene Expression Profile of Candidate Genes

**FIGURE 5:**
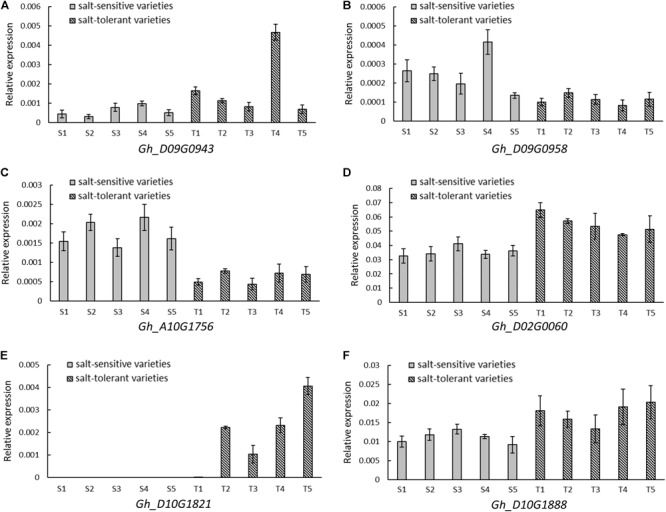
Relative expression by quantitative RT-PCR of six candidate genes significantly associated with RSR and STL. **(A)** *Gh_D09G0943* (NAC090). **(B)** *Gh_D09G0958* (LEA). **(C)** *Gh_A10G1756* (CPK1). **(D)** *Gh_D02G0060* (CIPK2). **(E)** *Gh_D10G1821* (UPL6). **(F)** *Gh_D10G1888* (SINAT2). Five salt-sensitive varieties, S1: Pengze70, S2: Shannmian10, S3: Lumianyan35, S4: Jiwu2833, S5: Xinluzao38; five salt-tolerant varieties, T1: Zhongwu1038, T2: CCRI20, T3: Zhongkang5, T4: Litai8, T5: Zhongwu3385.

A total of 35 and 26 candidate genes were within the LD decay region on D09 and D10, respectively (Supplementary Tables S3, S4). *Gh_D09G0943* and *Gh_D09G0958* were close to the peak SNP i46598Gh at 34.83–35.22 Mb. *Gh_D10G1888* was located near the peak SNP i20955Gh at 52.62–52.80 Mb. Another three genes (*Gh_A10G1756, Gh_D02G0060*, and *Gh_D10G1821*) were located near the association peak of other chromosomes. The expression levels of these six genes were measured using five salt-tolerant varieties and five salt-sensitive varieties by qRT-PCR. In comparison to the salt-sensitive varieties, *Gh_D10G1888, Gh_D02G0060, Gh_D09G0943*, and *Gh_D10G1821* had a higher relative expression in the salt-tolerant varieties. Notably, the relative expression of *Gh_D10G1821* was high in the salt-tolerant varieties, but almost no expression was found in the salt-sensitive varieties. However, the other two genes, *Gh_D09G0958* and *Gh_A10G1756*, had higher expression in the salt-sensitive varieties (**Figure 5**). This provides further evidence that the six putative genes were closely associated with salt tolerance.

**FIGURE 6:**
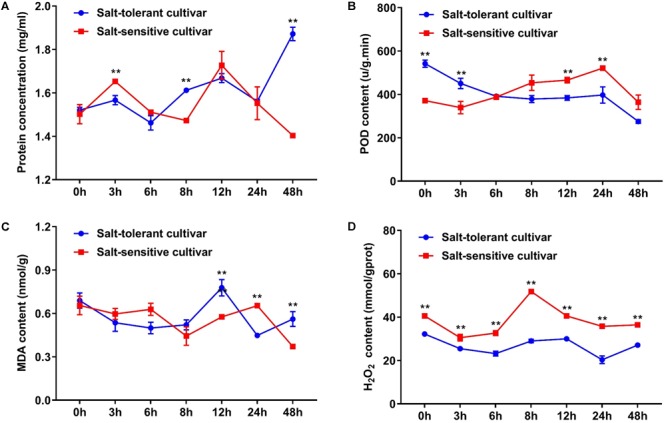
Comparison of the protein, POD, MDA, and H_2_O_2_ contents between the salt-tolerant cultivar and the salt-sensitive cultivar under salt stress. **(A)** Protein concentration. **(B)** POD content. **(C)** MDA content. **(D)** H_2_O_2_ content. ^∗∗^ indicates significance at *P* < 0.01 level (two-tailed *t*-test). Error bars indicate ± SD; *n* = 3 independent biological replicates.

In addition, we selected the two cultivars with significant difference of morphological characteristics under salt treatment for subsequent analysis. The contents of protein, POD, MDA, and H_2_O_2_ were used as physiological and biochemical indexes to characterize the tolerance to salt stress. Protein content increased gradually in the salt-tolerant cultivar and was significantly higher in the salt-tolerant cultivar than in the salt-sensitive cultivar at 48 h. POD content reached a peak at 3 h under salt treatment in the salt-tolerant cultivar; however, the POD content showed an upward tendency and continued to 24 h, where it reached a peak in the salt-sensitive cultivar. The MDA content totally decreased in the two types of cultivars, but showed a peak at 12 and 24 h. The H_2_O_2_ content increased in the salt-sensitive cultivar, however, it was basically unchanged in the salt-tolerant cultivar (**Figure 6**). These results indicated that there were differences in salt tolerance mechanisms between the two types of cultivars.

## Discussion

In the present study, we conducted a GWAS of salt-tolerant related traits at the seedling stage with a great many upland cotton accessions and SNPs from the CottonSNP63K array. This study provides new insights into the genetic basis of salt tolerance and the identification of the novel alleles underlying the variation in the salt-tolerant traits and candidate genes, allowing for accelerating the progress of cotton tolerance breeding.

The seedling stage is a very important stage in the cotton growth period. The RSR is the percentage of surviving seedlings divided by the total number of seeds used in the test, which is a critical feature to evaluate the seedling quality and viability. STL is considered a reliable index for evaluating salt tolerance on the corresponding seedling survival rate. In our study, therefore, both RSR and STL were selected to evaluate the upland cotton seedlings’ ability to salt stress. The seedling stage of cotton is about 10 days in the field and slightly shorter in the chamber; thus, we chose 7 days to investigate the two traits (Wang et al., 2011). Du et al. (2016) performed the evaluation of salt tolerance at the germination and seedling stages of 304 upland cotton cultivars and identified 43 advanced salt-tolerant cultivars by cluster analysis of 10 salt-tolerance related traits. Of the 713 accessions in this study, 142 were identified as salt-tolerant cultivars, 17 were salt-resistant cultivars and one accession had a high salt-resistance level. In addition, we found that the obsolete cultivars or lines were more salt-tolerant than the newly bred ones (Supplementary Table S1). This is perhaps because salt tolerance was gradually lost when breeders focused on high yield and improved fiber quality. In China, the planting area for the cotton already reduced greatly when agricultural industrial restructuring, and breeders are trying to develop and utilize saline-alkali land suitable for farming. However, because of the lack of excellent improved salt-tolerant cotton varieties, salinity is a serious constraint to cotton productivity in such areas. Therefore, these better salt-tolerant accessions could be selected as parents to accelerate the progress of cotton tolerance breeding by molecular design (Borsani et al., 2003; Rai et al., 2011).

Salt tolerance is a genetically and physiologically complex trait controlled by multiple small effect genes (Flowers, 2004). With the fast development of SNP arrays and next-generation sequencing technology, GWAS is becoming a novel and effective method for determining useful genes in crop plants. Association analysis has been successfully used in mining candidate genes of important agronomic traits in cotton, such as fiber quality, yield and its components, and Verticillium wilt resistance (Su et al., 2016a,b; Fang et al., 2017; Li et al., 2017; Sun et al., 2017; Wang et al., 2017). However, only a few studies related to salt tolerance were reported with natural populations and genome-wide molecular markers using the GWAS strategy. Jia et al. (2014a) only detected three SSR markers associated with salt tolerance in MLM using 106 SSRs in 323 *G. hirsutum* germplasms. Cai et al. (2017) found 9 intron length polymorphisms (ILPs) markers for 10 salt stress traits with 535 ILP markers in 264 *G. hirsutum* accessions. In this study, 713 upland cotton accessions were used to conduct a GWAS of two salt-tolerance related traits with 10,511 SNPs, and we identified 10 and 15 loci for RSR and STL, respectively, of which two common SNP loci (i46598Gh and i47388Gh) on D09 were significantly associated with the two traits. Perhaps this chromosome has important genomic hotspots controlling salt tolerance similar to other traits (Said et al., 2015). Furthermore, we screened 280 possible candidate genes that were involved in several kinds of functional proteins including transcription factors, transporters and enzymes. However, little is known about the genetic architecture of salt tolerance in cotton. These associated SNPs and candidates remain to be further verified and discussed in future research. Currently, many studies have dissected the genetic mechanisms of salt tolerance in Arabidopsis and other crop species, and many important candidate genes and pathways for salinity tolerance have been identified in *Arabidopsis thaliana* (Zhu, 2001; Ashraf et al., 2013; Deinlein et al., 2014).

On chromosome D09, the two genes *Gh_D09G0943* and *Gh_D09G0950* encode NAC domain-containing proteins. NAC proteins have received much attention as regulators in various stress signaling pathways, which may include the interplay of phytohormones (Puranik et al., 2012). It was demonstrated that *ONAC022* improved drought and salt stress tolerance through modulating an ABA-mediated pathway in rice (Hong et al., 2016). Two NAC transcription factors from *Caragana intermedia* altered ABA signaling during seed germination and enhanced salt tolerance of transgenic Arabidopsis (Han X. et al., 2015). Late embryogenesis abundant (LEA) proteins participated in tolerance to salinity and drought in many different organisms. We identified two candidate genes *Gh_D09G0958* and *Gh_D09G0959*, encoding LEA proteins. The LEA proteins could act as membrane stabilizers to prevent cellular collapse (Wang et al., 1996). In rice, the overexpression of *OsLEA4* in transgenic plants conferred increased resistance to salt, drought and heavy metal stresses (Hu et al., 2016). In addition, five homogenous genes located on different chromosomes belonged to pentatricopeptide repeat (PPR) proteins family, of which two (*Gh_D09G0932* and *Gh_D09G0961*) were located on D09. PPR proteins are considered to play important role in photosynthesis, plant development, and environmental responses (Barkan and Small, 2014). It was reported that the overexpression of a PPR gene improved salt tolerance in Arabidopsis (Zsigmond et al., 2012).

For RSR, the gene *Gh_A10G1756* encodes a calcium-dependent protein kinase (CPK or CDPK). In rice, the overexpression of *OsCPK4* positively regulated salt and drought tolerance via protection against membrane lipid peroxidation (Campo et al., 2014). On chromosome D08, the gene *Gh_D08G1976* coded for a homeobox-leucine zipper protein (HB protein). The ectopic expression of *HaHB1* from sunflowers in Arabidopsis conferred tolerance to drought and salinity stresses by the induction of proteins that stabilize membranes (Cabello and Chan, 2012). There is another gene, *Gh_D08G1982*, encoding a DEAD-box helicase protein. A DEAD-box helicase, PDH45, conferred salinity tolerance to rice in both the seedling and reproductive stage (Amin et al., 2011). Additionally, the four genes *Gh_D11G0691, Gh_D11G0694, Gh_D11G0702*, and *Gh_D11G0706* on chromosome D11 are homologous genes of PP2A, MYB40, WD40, and ZFP in Arabidopsis, respectively. Protein phosphatase 2A (PP2A) was one of the major serine/threonine protein phosphatases and played important roles in cellular processes in plants (Farkas et al., 2007). In Arabidopsis, PP2A-C5 increased salt tolerance in a pathway different from the SOS signaling pathway (Hu et al., 2017). Plant MYB transcription factors control diverse biological processes. In birch, BplMYB46 improved salt and osmotic stress tolerance and mediated secondary cell wall deposition (Guo et al., 2017). A wheat WD40 repeat-containing protein increased plant tolerance to ABA, salt stress and osmotic stress during seed germination and seedling development (Kong et al., 2015). In cotton, GhZFP1, a novel CCCH-type zinc finger protein from cotton, acted as a novel positive regulator to confer salt tolerance and fungal pathogen resistance to plants (Guo et al., 2009).

For STL, the three genes *Gh_D10G1821, Gh_D10G1842*, and *Gh_D10G1888* encode the E3 ubiquitin-protein ligase. E3 ubiquitin was involved in the response to dehydration stress and the regulation of proline biosynthesis in Arabidopsis (Yee and Goring, 2009). The Arabidopsis U-box E3 ubiquitin ligase AtPUB30 participated in the salt stress tolerance as a negative factor during the germination stage (Hwang et al., 2015). There are two genes, *Gh_D02G0060* and *Gh_D10G1874*, that encode CBL-interacting protein kinase (CIPK). *CaCIPK6* increased auxin transport and hypersensitivity to auxin and promoted salt tolerance in transgenic tobacco (Tripathi et al., 2009). The overexpression of *TaCIPK25* impaired salt tolerance, which was mediated by a WRKY transcription factor in an ABA-dependent pathway under saline conditions (Jin et al., 2016). The gene *Gh_D10G1824* encoded a MATE efflux family protein that modulates ABA sensitivity and increases tolerance to drought with lower stomatal conductance (Zhang et al., 2014). Another gene *Gh_A01G0243*, encodes an Na^+^/H^+^ antiporter. It was responsible for the regulation of internal pH, cell volume and sodium level in the cytoplasm (Bassil et al., 2011). Cotton*GhNHX1* functioned as a tonoplast Na^+^/H^+^ antiporter and played an important role in the salt tolerance of cotton (Wu et al., 2004).

## Conclusion

The cotton accessions consisting of diverse germplasms worldwide showed large variations in RSR and STL under salt stress. A total of 10 and 15 SNPs significantly associated with RSR and STL were identified, respectively, of which the two SNPs i46598Gh and i47388Gh on D09 were simultaneously associated with the two traits. We further screened 280 candidate genes, many of which were related to plant salt tolerance. In addition, we validated the expression level of six putative genes using five salt-tolerant and five salt-sensitive varieties under salt stress by qRT-PCR. Our results provided a valuable reference for the study of salt tolerance in *G. hirsutum*. These SNP loci and candidate genes would be useful for future salt tolerance breeding programs in cotton.

## Author Contributions

ZM, GZ, and XW conceived and designed the research. ZS, HL, YZ, ZL, HK, and LW performed the experiments. ZS and HL conducted statistical analysis of data. ZS and XW wrote the manuscript. ZM revised the manuscript.

## Conflict of Interest Statement

The authors declare that the research was conducted in the absence of any commercial or financial relationships that could be construed as a potential conflict of interest.

## Footnotes

1 https://cottonfgd.org/
